# On nonstandard finite difference schemes in biosciences

**DOI:** 10.1063/1.4758961

**Published:** 2012-10-02

**Authors:** R. Anguelov, Y. Dumont, J. M.-S. Lubuma

**Affiliations:** Department of Mathematics and Applied Mathematics, University of Pretoria, South Africa; CIRAD, Umr AMAP, Montpellier, France; Department of Mathematics and Applied Mathematics, University of Pretoria, South Africa; Technical University of Sofia, Sofia, Bulgaria

## Abstract

We design, analyze and implement nonstandard finite difference (NSFD) schemes for some differential models in biosciences. The NSFD schemes are reliable in three directions. They are topologically dynamically consistent for onedimensional models. They can replicate the global asymptotic stability of the disease-free equilibrium of the MSEIR model in epidemiology whenever the basic reproduction number is less than 1. They preserve the positivity and boundedness property of solutions of advection-reaction and reaction-diffusion equations.

### INTRODUCTION

The ever growing complexity of natural processes leads to challenging mathematics for the analysis of models in biosciences. Even in those cases when the mathematical theory is well-established, the differential models cannot be completely solved by analytical techniques. Consequently, numerical simulations are of fundamental importance in gaining some useful insights on the solutions of the differential equations.

In recent years, the authors made important contributions on the design, analysis and implementation of nonstandard finite difference (NSFD) schemes in biosciences. However, the results were written in diverse contexts and are to some extent scattered in the literature. The purpose of this paper is to provide a unified and focused presentation that could be user friendly. (For works devoted to the applications of NSFD schemes, we can mention the edited volumes [1, 2] and the references therein).

We report on reliable numerical schemes for bio-models in the following three directions/sections: One-dimensional models, whereby NSFD schemes that are topologically dynamically consistent are considered; Epidemiological models, whereby the focus is on NSFD schemes that replicate the global asymptotic stability of the disease-free equilibrium; Advection-reaction-diffusion models, whereby the interest is in NSFD schemes that preserve the positivity and the boundedness property of solutions. The last section is devoted to concluding remarks where potential research problems for future work are mentioned.

### ONE-DIMENSIONAL DYNAMICAL SYSTEMS

In biosciences, simple models, specifically one-dimensional ones, constitute a convenient setting for developing, demonstrating and testing new methods and approaches, which then can be extended to multidimensional systems. Here, we restrict ourselves to three important examples. We start with the decay equation

$$\frac{du}{dt} = -ay, \quad u(0) = u^0 \quad a > 0, \tag{1}$$

[1] Corresponding author

*Application of Mathematics in Technical and Natural Sciences*
AIP Conf. Proc. 1487, 212-223 (2012); doi: 10.1063/1.4758961
© 2012 American Institute of Physics 978-0-7354-1099-2/$30.00

which arises in many applied settings such as population dynamics and compartmental models in epidemiology. The solution of the decay equation is

$$u(t) = u^0 e^{-at} \tag{2}$$

and, thus, the corresponding exact scheme is [3]:

$$\frac{u^{k+1} - u^k}{(1 - e^{-ah})/a} = -au^k \text{ or } \frac{u^{k+1} - u^k}{(e^{ah} - 1)/a} = -au^{k+1}. \tag{3}$$

Here and after, for $k = 0, 1, 2, \cdots$, $u^k$ denotes an approximation to the solution $y(t)$ at the discrete time $t = t_k = kh$ where $h = \Delta t$ is the step size. The second example is the logistic equation

$$\frac{du}{dt} = au(1-u), \;\; u(0) = u^0, \tag{4}$$

which is abundantly used in biosciences. Its solution is

$$u(t) = \frac{u^0}{u^0 + (1-u^0)e^{-at}}, \tag{5}$$

which yields the exact scheme [3]:

$$\frac{u^{k+1} - u^k}{(e^{ah} - 1)/a} = au^k(1 - u^{k+1}). \tag{6}$$

As a third example, we consider a less trivial one, namely the Michaelis-Menten (M-M) model:

$$\frac{du}{dt} = -\frac{au}{1+bu}, \; u(0) = u^0; \, a > 0, \; b \geq 0. \tag{7}$$

Its exact scheme, constructed recently in terms of the Lambert $W$ function, is [4]:

$$\frac{u^{k+1} - u^k}{(1 - e^{-ah})/a} = \frac{W\left((b\exp(-ah)u^k \exp(bu^k)\right) - bu^k}{b(1 - e^{-ah})/a}. \tag{8}$$

More generally, we consider a dynamical system on an interval $D \subset \mathbb{R}$ defined by a scalar differential equation

$$\frac{du}{dt} = f(u), \;\; u(0) = u^0, \tag{9}$$

as well as a numerical scheme of the form

$$u^{k+1} = G(h; u^k), \tag{10}$$

which is a discrete dynamical system on $D$. Obviously, the function $G = G(h) = G(h; y)$ must satisfy the usual consistency conditions for the argument $u$:

$$G(0; u) = u \qquad \text{and} \qquad \frac{dG(0)}{dh}(u) = f(u). \tag{11}$$

It is also assumed that involved functions have the needed smoothness properties. The exact schemes of our three examples and in fact the exact schemes of the many models investigated in [3, 5] involve two characteristics that led two of the authors [6] to the following definitions:

**Definition 1** *1. A finite difference equation (10) that determines approximate solutions $u^k$ to the solution $u(t)$ of a differential equation (9) is called a nonstandard finite difference (NSFD) scheme if at least one of the following two conditions is met:*

- *The classical denominator $h$ of the discrete derivative is replaced by a nonnegative function $\phi(h)$ such that*

$$\phi(h) = h + O(h^2) \text{ as } h \to 0; \tag{12}$$

- *Nonlinear terms that occur in the right-hand side $f(u)$ of the differential equation (9) are approximated in a nonlocal way, i.e., by a suitable function of several points of the mesh.*

2. *A NSFD scheme is called (qualitatively) stable or dynamically consistent with respect to some property P of the differential equation, whenever the discrete equation replicates the property P for every value of h.*

To go further with the qualitative feature of NSFD schemes, we assume that the following condition holds for all arguments $h$ and $u$:

$$\frac{dG(h;u)}{du} > 0. \tag{13}$$

We have the following result [7]. The first part is an important "practical" characterization of Condition (13), while the second part is a substitute for elementary stability in the case of non-hyperbolic fixed points.

**Theorem 2** *The condition* (13) *is necessary and sufficient for the scheme* (10) *to be qualitatively stable with respect to monotone dependence on the initial value,* i.e.*,*

$$u^0 < v^0 \Longrightarrow u^k < v^k, \qquad k = 1,2,3,\ldots \tag{14}$$

*Furthermore, under the condition* (13)*, provided that the scheme* (10) *has no spurious fixed-points compared with the equilibrium points of the ODE* (9)*, this scheme is qualitatively stable with respect to the property of monotonicity of solutions in the sense that the sequence* $(u^k)$ *is increasing or decreasing when the exact solution* $u(t)$ *is increasing or decreasing, respectively.*

Theorem 2 makes the relevance of the condition (13) clear. This is further strengthen in the following precise manner [8]:

**Theorem 3** *Let* $D = [a,b] \subset \mathbb{R}$ *and let* $G \in C^1([0,\infty), C^1(D,D))$ *satisfy (11) and (13). Assume that for every* $h > 0$ *the fixed points of the map* $G(h) : D \to D$ *are the roots of* $f(u) = 0$*. Then the numerical scheme (10) is topologically dynamically consistent with the continuous dynamical systems (9) in the following sense: every map* $G(h)$ *is topologically equivalent to each evolution operator* $S(t)$ *of the dynamical system (9).*

In the light of Theorem 3, Condition (13) is the key requirement if numerical schemes are to replicate topological properties of the continuous dynamical systems. Coming back to our examples, we add to their exact schemes some NSFD schemes to which Theorem 3, including Condition (13), applies. For the logistic equation, we have the NSFD scheme [3]:

$$\frac{u^{k+1} - u^k}{h} = au^k(1 - u^{k+1}). \tag{15}$$

For the M-M model, we mention the following NSFD schemes [9, 10]:

$$\frac{u^{k+1} - u^k}{(e^{ah} - 1)/a} = -\frac{au^{k+1}}{1 + bu^k}; \tag{16}$$

$$\frac{u^{k+1} - u^k}{(e^{ah} - 1)/a} = -\frac{au^{k+1}}{1 + bu^{k+1}}; \tag{17}$$

$$\frac{u^{k+1} - u^k}{(1 - e^{-ah})/a} = -\frac{au^k}{1 + bu^k}. \tag{18}$$

Despite its apparent implicit nature, the NSFD scheme (17) is a discrete dynamical system on the interval $D = [0,\infty)$ that admits the representation (10) and meets the requirement (13), since this scheme is equivalent to a quadratic equation in $u^{k+1}$ that admits only one nonnegative root whenever $u^k \geq 0$.

In contrast, the forward Euler method fails to meet the requirement (13) when applied to the decay, the logistic and the M-M equations. The following figures speak for themselves as far as the performance of the NSFD and standard schemes is concerned: Figure 1 for the decay equation and Figure 2 for the logistic equation with $a = 1$ and $h = 1.8$; Figure 3 for the M-M equation where on each picture, the three NSFD schemes (16)-(18) for $b = 1$ and $b = 0$ (decay equation) are compared to the exact scheme (8).

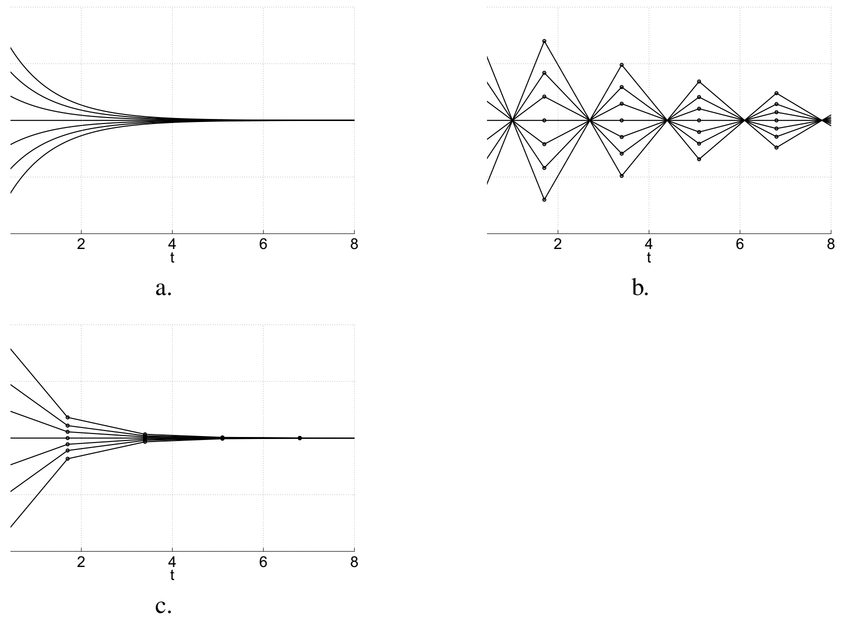

**FIGURE 1.** Decay equation (1): (a) Exact solution; (b) Forward Euler method; (c) Exact scheme

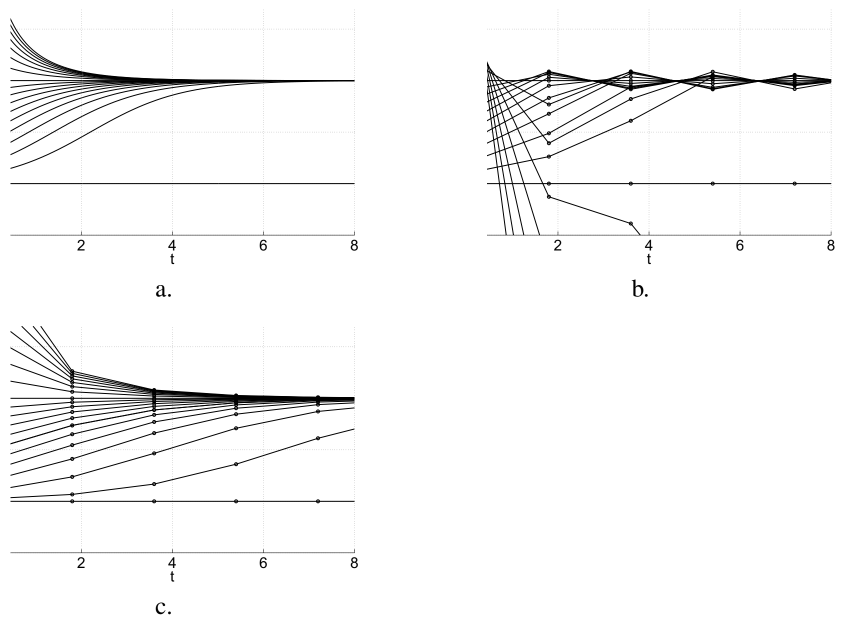

**FIGURE 2.** Logistic equation (4): (a) Exact solution; (b) Forward Euler method; (c) Exact scheme

## COMPARTMENTAL MODELS IN EPIDEMIOLOGY

We consider a class of dynamical systems on $\Omega \subset \mathbb{R}^n_+$ of the form

$$\frac{dx}{dt} = A(x)x + f, \quad x(0) = x^0, \tag{19}$$

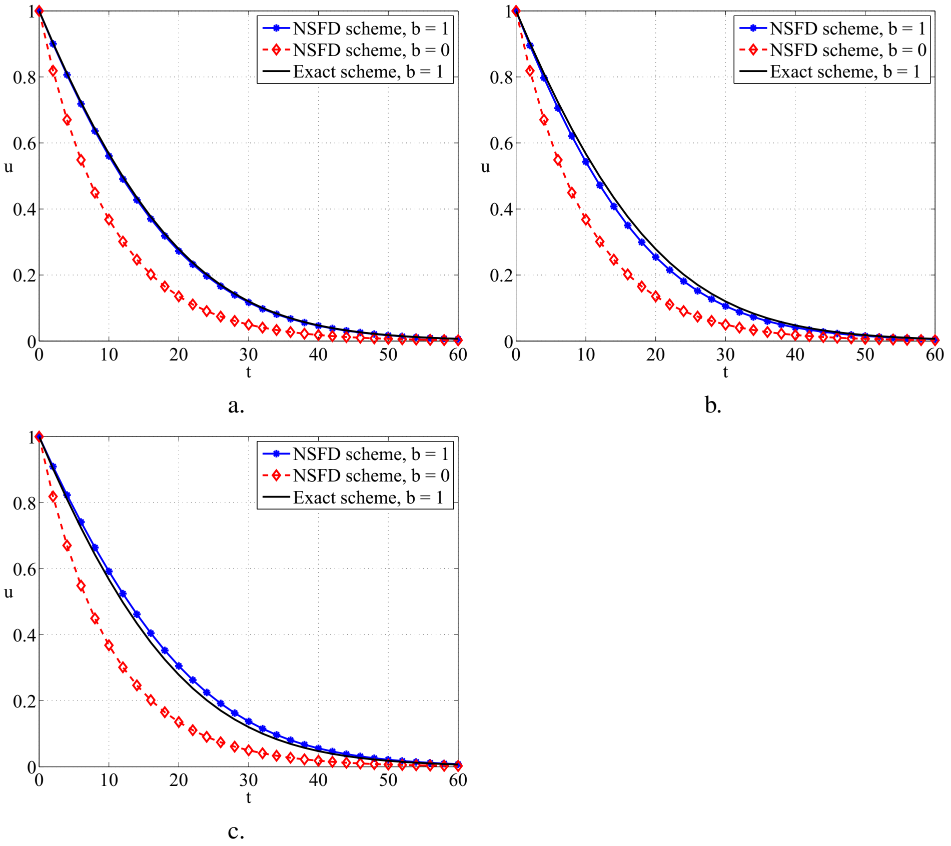

**FIGURE 3.** M-M equation (7): Exact scheme (8) versus NSFD schemes (16) for (a), (17) for (b) and (18) for (c)

where $x = x(t) : [0,\infty) \rightarrow \mathbb{R}^n$ represents the number/density of the populations, $x^0 \in \mathbb{R}^n$ represents the initial populations, some coordinates of the vector $f$ could be the recruitment/birth rates and $A(x)$ is a nonlinear real $n \times n$ Metzler matrix.

We make two typical assumptions. These are: the population $x$ is decomposable into non-infected $y$ and infected $z$ individuals, *i.e.*, $x = (y,z) \in \mathbb{R}^{n_1} \times \mathbb{R}^{n_2}$, with $n_1 + n_2 = n$; there exists a unique disease-free equilibrium (DFE) of the form $x^* = (y^*, 0)$, $y^* \neq 0$.

We are interested in the global asymptotical stability (GAS) of the DFE, a property that is highly desirable in order to design an intervention to reduce the spread of disease or to eradicate it. More precisely, we study numerical schemes for (19) that are reliable in that they replicate the GAS of the DFE. The schemes under consideration are discrete dynamical systems on $\Omega$ of the following form having the disease-free equilibrium $x^*$ as fixed-point:

$$x^{k+1} = F(x^k). \tag{20}$$

Checking the GAS of an equilibrium/fixed-point is usually based on a Lyapunov function, which is unfortunately not easy to construct. Thus, we use an alternative approach that is based on the availability of the following representations of the systems (19) and (20):

$$\begin{cases} \frac{dy}{dt} = A_1(y,z)(y - y^*) + A_{12}(y,z)z, \\ \frac{dz}{dt} = A_2(y,z)z. \end{cases} \tag{21}$$

$$\begin{cases} y^{k+1} = A_1\left(y^k, z^k\right)\left(y^k - y^*\right) + A_{12}\left(y^k, z^k\right) z^k, \\ z^{k+1} = A_2\left(y^k, z^k\right) z^k. \end{cases} \tag{22}$$

Here $A_1(x)$, $A_2(x)$ and $A_{12}(x)$ are $n_1 \times n_1$, $n_2 \times n_2$ and $n_1 \times n_2$ matrices that need not be the same for the discrete and the continuous systems.

For the continuous system, the result due to [11] reads as follows:

**Theorem 4** *Assume that*

$H_1$. *The dynamical system (21) or (19) is dissipative on* $\Omega$*;*

$H_2$. *The equilibrium point* $y^*$ *of the sub-system*

$$\dot{y} = A_1(x)(y - y^*) \tag{23}$$

*is GAS on the canonical projection of* $\Omega$ *on* $\mathbb{R}_+^{n_1}$*;*

$H_3$. *The matrix* $A_2(x)$ *is a Metzler matrix and is irreducible for each* $x \in \Omega$*;*

$H_4$. *There exists an upper bound matrix* $\bar{A}_2$ *for the set* $\mathscr{M} = \{A_2(x) : \quad x \in \Omega\}$ *with the following property: either* $\bar{A}_2 \notin \mathscr{M}$*, or* $\bar{A}_2 \in \mathscr{M}$ *and for each* $\bar{x} \in \Omega$ *satisfying* $\bar{A}_2 = A_2(\bar{x})$*, we necessarily have* $\bar{x} \in \mathbb{R}_+^{n_1} \times \{0\}$*;*

$H_5$. $\alpha\left(\bar{A}_2\right) \leq 0$*, where* $\alpha\left(\bar{A}_2\right)$ *is the maximum of the real parts of eigenvalues of the matrix* $\bar{A}_2$*.*

*Then, the disease-free equilibrium* $x^*$ *is GAS in* $\overline{\Omega}$*.*

The discrete counterpart of Theorem 4 is established in [12]:

**Theorem 5** *Assume that*

$D_1$. *The discrete dynamical system (22) or (20) is dissipative on* $\Omega$*;*

$D_2$. *The fixed-point* $y^*$ *of the sub-system*

$$y^{k+1} = A_1\left(y^k, 0\right)(y^k - y^*)$$

*is GAS on the set* $\Omega_1 = \{y \in \mathbb{R}^{n_1} : (y,0) \in \Omega\}$*;*

$D_3$. *The matrix* $A_2(y,z)$ *is nonnegative for all* $(y,z) \in \Omega$*;*

$D_4$. *There exists an irreducible matrix* $\overline{A}_2$*, which is an upper bound for the set* $\mathscr{M} = \{A_2(y,z) : (y,z) \in \Omega\}$.

$D_5$. *The matrix* $\overline{A}_2$ *is such that its spectral radius satisfies* $\rho(\overline{A}_2) \leq 1$*. In the case when* $\rho(\overline{A}_2) = 1$*, we assume that* $A_2(y,z) >> 0$, $\forall (y,z) \in \Omega$ *and, in addition, z reduces to the zero vector in* $\mathbb{R}^{n_2}$ *if there exists a vector* $(y,z) \in \Omega$ *such that* $A_2(y,z) = \overline{A}_2$*.*

*Then, the fixed-point* $(y^*, 0)$ *is GAS for discrete system on* $\Omega$*.*

Theorems 4 and 5 apply to a variety of epidemiological models (see for example [11, 13, 14, 15]). To be more specific, we consider the MSEIR epidemiological model that involves a total population of size $N = N(t)$ divided in five compartments: $M = mN$ - infants with passive immunity, $S = sN$ - susceptible, $E = eN$ - exposed, $I = iN$ - infective, and $R = rN$ - recovered individuals. Figure 4 shows the flow and the corresponding constant rates between compartments, with $b$ and $d$ being the recruitment and death rates, respectively.

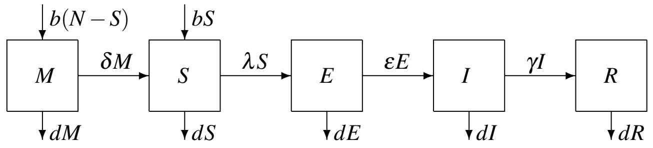

**FIGURE 4.** Flow diagram of the MSEIR model

With $q := b - d$, the corresponding equations of the MSEIR model are

$$\frac{dx}{dt} = A(x)x \ \text{ or } \ \begin{cases} \dfrac{dm}{dt} &= (d+q)(e+i+r) - \delta m, \\ \dfrac{ds}{dt} &= -\beta si + \delta m, \\ \dfrac{de}{dt} &= \beta si - (\varepsilon + d + q)e, \\ \dfrac{di}{dt} &= \varepsilon e - (\gamma + d + q)i, \\ \dfrac{dr}{dt} &= \gamma i - (d+q)r, \end{cases} \tag{24}$$

where $x=(m,s,e,i,r)^T$ and the compartmental matrix is given by

$$A(x)=\begin{pmatrix} -\delta & 0 & (d+q) & (d+q) & (d+q)\\ \delta & -\beta i & 0 & 0 & 0\\ 0 & \beta i & -(\varepsilon+d+q) & 0 & 0\\ 0 & 0 & \varepsilon & -(\gamma+d+q) & 0\\ 0 & 0 & 0 & \gamma & -(d+q) \end{pmatrix}.$$

The disease-free equilibrium is $DFE\equiv x^*=(0,1,0,0,0)^T$. On setting

$$\Omega=\{(m,s,e,i,r)\in\mathbb{R}^5_+:\quad m+s+e+i+r=1\}, \tag{25}$$

$x=(y,z)^T$, $y=(m,s,r)^T$, $z=(e,i)^T$, $y^*=(0,1,0)^T$,

$$A_1(x)=\begin{pmatrix} -\delta & 0 & (d+q)\\ \delta & -\beta i & 0\\ 0 & 0 & -(d+q) \end{pmatrix},\quad A_{12}=\begin{pmatrix} d+q & d+q\\ 0 & -\beta\\ 0 & \gamma \end{pmatrix},$$

and

$$A_2(x):=\begin{pmatrix} -(\varepsilon+d+q) & \beta s\\ \varepsilon & -(\gamma+d+q) \end{pmatrix}\le\begin{pmatrix} -(\varepsilon+d+q) & \beta\\ \varepsilon & -(\gamma+d+q). \end{pmatrix}=:\overline{A}_2$$

for $x\in\Omega$, it follows that the model (24) admits the matrix representation (21). More so, the requirements (H1)-(H4) in Theorem 4 are met, the requirement (H5) being equivalent to (26) below. Thus, we rediscover the following known result [16]:

**Theorem 6** *For MSEIR model, a dissipative dynamical system on the biologically feasible region $\Omega$ in (25), the DFE is GAS whenever the basic reproduction number is less than 1:*

$$\mathscr{R}_0:=\frac{\beta\varepsilon}{(\varepsilon+d+q)(\gamma+d+q)}\le 1. \tag{26}$$

Given $\theta\in[0,1]$ and $\hat\theta\in[0,1]$, we consider the NSFD scheme:

$$\begin{aligned}
\frac{m^{k+1}-m^k}{\phi(h)} &= -\delta[\theta m^{k+1}+(1-\theta)m^k]+\theta(d+q)(r^{k+1}+e^{k+1}+i^{k+1})\\
&\quad +(1-\theta)(d+q)(r^k+e^k+i^k)\\
\frac{s^{k+1}-s^k}{\phi(h)} &= -\beta[\hat\theta s^{k+1}i^k+(1-\hat\theta)s^k i^{k+1}]+\delta[\theta m^{k+1}+(1-\theta)m^k]\\
\frac{e^{k+1}-e^k}{\phi(h)} &= -(\varepsilon+d+q)[\theta e^{k+1}+(1-\theta)e^k]+\beta(\hat\theta s^{k+1}i^k+(1-\hat\theta)s^k i^{k+1})\\
\frac{i^{k+1}-i^k}{\phi(h)} &= \varepsilon[\theta e^{k+1}+(1-\theta)e^k]-(\gamma+d+q)[\theta i^{k+1}+(1-\theta)i^k]\\
\frac{r^{k+1}-r^k}{\phi(h)} &= -(d+q)[\theta r^{k+1}+(1-\theta)r^k]+\gamma[\theta i^{k+1}+(1-\theta)i^k].
\end{aligned}$$

The denominator function $\phi(h)$, chosen in accordance with (12), must satisfy some additional conditions in order to guarantee the following important facts :

- The NSFD scheme amounts to solving at each step a linear system

$$\tilde C(i^k,s^k)\begin{pmatrix} m^{k+1} & s^{k+1} & r^{k+1} & e^{k+1} & i^{k+1}\end{pmatrix}^T=\tilde D\begin{pmatrix} m^k & s^k & r^k & e^k & i^k\end{pmatrix}^T, \tag{27}$$

where $\tilde C(i^k,s^k)$ is an $M$-matrix, $\tilde D\ge 0$ and thus the scheme is a dissipative discrete dynamical system on $\Omega$;
- The NSFD scheme is second-order convergent when $\theta=\hat\theta=1/2$;

• The NSFD scheme satisfies the conditions in Theorem 5, specifically (D3), (D4) and (D5).

Typically, as illustrated in the numerical examples below, we take

$$\phi(h) = \varphi(hQ)/Q \text{ with } 0 < \varphi(h) < 1, \tag{28}$$

where the number $Q$ captures the feature of the model through the condition

$$Q \geq (1-\theta)\max\{(1-\theta)\delta, (1-\theta)(\varepsilon+d+q), (1-\theta)(\gamma+d+q), (1-\hat{\theta})\beta\}. \tag{29}$$

In summary, for such a suitable $\phi(h)$, we obtain the following result [12]:

**Theorem 7** *For $\theta$ and $\hat{\theta}$ such that either $\theta = 0$ or $\theta + \hat{\theta} \geq 1$, the fixed-point $x^* = (0,1,0,0,0)$ of the NSFD scheme, on the set $\Omega$, is GAS whenever $\mathscr{R}_0 < 1$. Furthermore, the NSFD scheme is second-order convergent if $\theta$=$\hat{\theta}$=1/2 and first-order convergent otherwise.*

**Remark 8** *As mentioned earlier, the authors have applied Theorems 4 and 5 to other compartmental models (see for instance [13, 14, 15]). The more critical part is to check the conditions (H5) and (D5). In general, this amounts to checking that the basic reproduction number satisfies the condition $\mathscr{R}_0 \leq 1$, as stated in (26). However, an additional threshold number $\xi < 1$ may arise so that the GAS of the DFE is rather subjected to a more restrictive condition $\mathscr{R}_0 \leq \xi$. For $\xi < \mathscr{R}_0 \leq 1$, we can have the backward bifurcation phenomenon whereby there is co-existence of the stable DFE with a stable endemic equilibrium. We investigated the backward bifurcation in [13] for a malaria model and in [17] for a disease, like the HIV/AIDS, with multiple strains.*

In the numerical simulations below, where $h = 2$ or $h = 10$, we use the following values: $\beta = 0.14$, $\delta = 1/180$, $\varepsilon = 1/14$, $\gamma = 1/7$, $d = 1/(40*365)$, $q = 0$ and thus $\mathscr{R}_0 = 0.97857$. While the theory on the GAS of the DFE is perfectly supported by Figure 5 on the NSFD scheme, Figure 6 displays so well the poor performance of classical methods to the extent that solutions can be negative. For the second-order convergence, the result on Figure 5 (d) are further tabulated in [12]. Note that in each figure, we plot the fractions of populations against days as time unit.

### ADVECTION REACTION DIFFUSION MODELS

The advection, reaction and diffusion processes are naturally involved in differential models in biosciences. We start with the two-dimensional advection-reaction equation

$$\begin{cases} u_t + au_x + bu_y &= r(u) \\ u(x,y,0) &= f(x,y) \end{cases} \tag{30}$$

where the reaction term $r(u)$, which is assumed to have a nonzero finite number of roots, and the initial function $f(x,y)$ are as smooth as needed, $a$ and $b$ being positive constants. Here we assume the following regarding the solution $u$ and the initial condition $f$:

$$0 \leq f \leq 1 \Longrightarrow 0 \leq u \leq 1. \tag{31}$$

We are then interested in constructing NSFD schemes that preserve the property (31). By the method of characteristics, the solution of (30) is:

$$u(x,y,t) = \begin{cases} f(x-at, y-bt)e^{\lambda t} & \text{if} \quad r(u) = \lambda u \\ \dfrac{f(x-at,y-bt)}{e^{-\lambda t} + (1-e^{-\lambda t})f(x-at,y-bt)} & \text{if} \quad r(u) = \lambda u(1-u). \end{cases} \tag{32}$$

This leads to the exact scheme [3]

$$\begin{cases} \dfrac{u^{k+1}_{m,n} - u^k_{m-1,n-1}}{(e^{\lambda\triangle t}-1)/\lambda} &= \lambda u^k_{m-1,n-1} & \text{if} \quad r(u) = \lambda u \\ \dfrac{u^{k+1}_{m,n} - u^k_{m-1,n-1}}{(e^{\lambda\triangle t}-1)/\lambda} &= \lambda u^k_{m-1,n-1}(1-u^{k+1}_{m,n}) & \text{if} \quad r(u) = \lambda u(1-u). \end{cases} \tag{33}$$

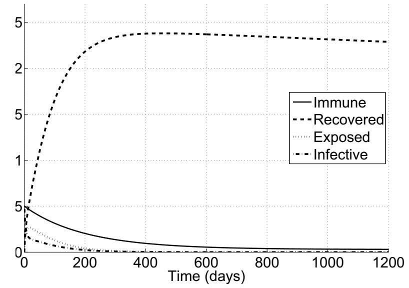

a. $\theta = 0$ and $\hat{\theta} = 1$ with $\phi(h) = (1 - e^{-Qh})/Q$,

$Q = \max\{\delta, \varepsilon + d + q, \gamma + d + q\}$

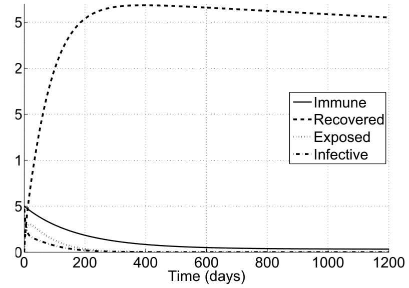

b. $\theta = \hat{\theta} = 1$ with $\phi(h) = h$

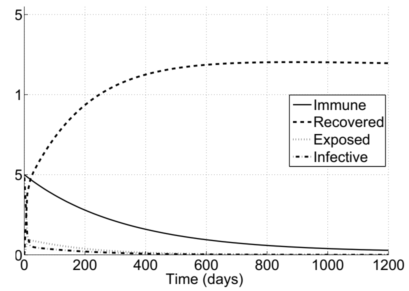

c. $\theta = \hat{\theta} = 0$ where $\phi(h) = (1 - e^{-Qh})/Q$,

$Q = \max\{\delta, \varepsilon + d + q, \gamma + d + q, \beta\}$

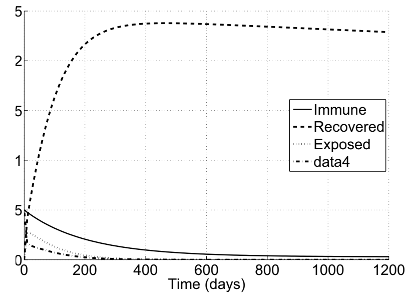

d. $\theta = \hat{\theta} = \frac{1}{2}$ with $\phi(h) = (\tanh(Qh))/Q$,

$Q = \max\{\delta, \varepsilon + d + q, \gamma + d + q, \beta\}/2$

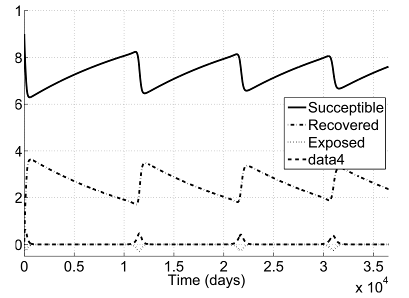

**FIGURE 5.** NSFD Scheme: (a) Explicit 1; (b) Implicit; (c) Explicit 2; (d) Second-order a.

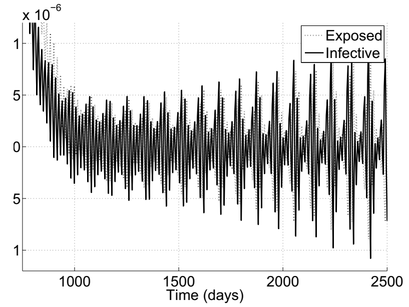

b.

**FIGURE 6.** Dynamical inconsistency of (a) 2nd order Runge-Kutta method and (b) Matlab routine ODE45

whenever the time and space step sizes satisfy the following functional relations:

$$\Delta x = a\Delta t \qquad \text{and} \qquad \Delta y = b\Delta t. \tag{34}$$

Here, $u^k_{m,n}$ denotes an approximation of $u(x,y,t)$ at the grid point $t = k\Delta t$, $x = m\Delta x$ and $y = n\Delta y$. Thus for a general reaction term, we can consider the NSFD scheme

$$\frac{u^{k+1}_{m,n} - u^k_{m-1,n-1}}{\phi(Q\Delta t)/Q} = r(u^k_{m-1,n-1}). \tag{35}$$

Similarly to (28) and (29), the feature of the model is captured by a number $Q \geq \max\{|r'(\widetilde{u})|; r(\widetilde{u}) = 0\}$ as well as by a function $\phi$ like in (12) that also satisfies

$$0 < \phi(z) < 1 \text{ for } z > 0. \tag{36}$$

It is proved in [18], that apart from being elementary stable, the NSFD scheme (35) preserves the property (31) whenever (34) holds and $-s \leq r(s)/Q \leq 1-s$ for $0 \leq s \leq 1$. This result is illustrated in Figure 7 for the numerical solution at time $t = 1$ with $r(u) = \lambda u(1-u)$, $\lambda = 10$, $f(x,y) = e^{-x^2-y^2}$, $a = b = 1$, $\Delta t = 0.2$ and $\phi(\Delta t) = (1 - e^{-10\Delta t})/10$. The poor performance of Lax's classical finite difference method (*i.e.*, the denominator in (35) is $\Delta t$) is also visualized.

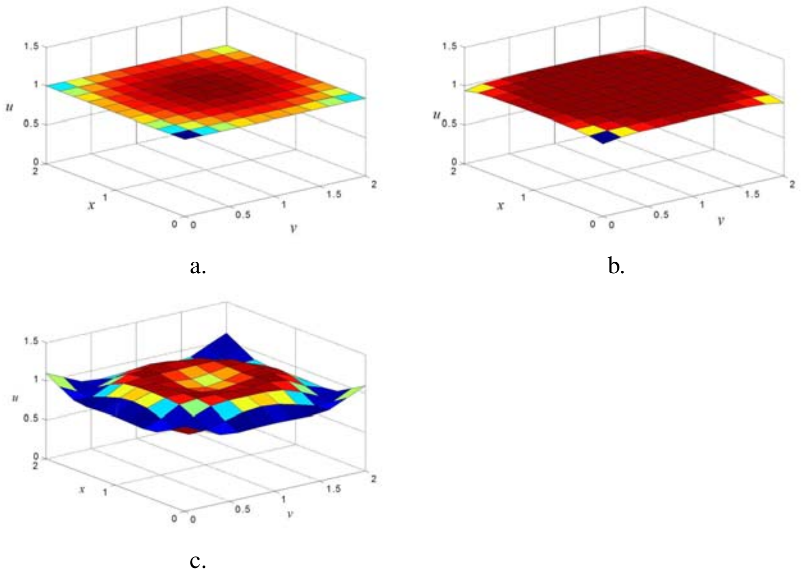

**FIGURE 7.** (a) Exact scheme ($33_2$), (b) NSFD scheme (35), (c) Lax scheme

Next, we consider the one-dimensional reaction diffusion equation

$$u_t = u_{xx} + r(u), \quad u(x,0) = f(x) \tag{37}$$

for which we make the assumption (31). NSFD schemes that preserve the property (31) for the general equation (37) are discussed in [19]. In the particular case of the Fisher equation

$$u_t = u_{xx} + \lambda u(1-u), \tag{38}$$

which satisfies the property (31) (see [20]), a NSFD scheme of the form

$$\frac{u_m^{k+1} - u_m^k}{\phi(\Delta t)} = \frac{u_{m+1}^k - 2u_m^k + u_{m-1}^k}{(\Delta x)^2} + \lambda(1 - u_m^{k+1})\frac{u_{m-1}^k + u_m^k + u_{m+1}^k}{3} \tag{39}$$

is proposed in [19] with various expressions of the denominator function $\phi(\Delta t)$. Further extension to the SIS-diffusion model is done in [21]. It is proved in [19] that the NSFD scheme (39) replicates the property (31) whenever we have the following functional relation between the step sizes:

$$2\phi(\Delta t) = (\Delta x)^2. \tag{40}$$

The result is illustrated on Figure 8 for the initial function $f(x) = 0.5 + 0.5\sin 2x$, $\lambda = 25$ and $\Delta x = .25$ for various $\phi(\Delta t)$. The pictures confirm the superiority of the NSFD schemes over the standard explicit finite difference scheme, which further displays unwanted oscillations.

NSFD schemes and other methods for the full advection-reaction-diffusion equation as well as their impact in biosciences are investigated in [9, 10, 22, 23].

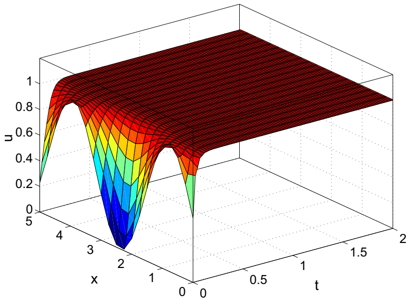

a. $\Delta t = 0.061$

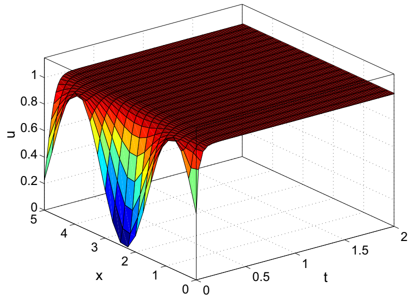

b. $\Delta t = 0.0231$

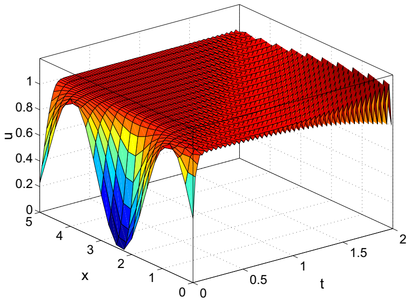

c. $\Delta t = 0.0231$

**FIGURE 8.** NSFD scheme with: (a) $\phi(\Delta t) = (1 - e^{-25\Delta t})/25$, (b) $\phi(\Delta t) = (e^{25\Delta t} - 1)/25$; Standard scheme: (c)

### CONCLUSION

We gave an overview of our recent work on NSFD schemes in biosciences, with the emphasis on three aspects of the reliability and excellence performance of these schemes. Each aspect of the study leads on its own to research problems on which we are currently working. These include: the extension of the concept of topologically dynamically consistent NSFD schemes to multi-dimensional models [8], the design of positivity-preserving NSFD schemes for cross diffusion equations (or other models in ecology) [24, 25, 26] and the construction of NSFD schemes that replicate the backward bifurcation phenomenon for Volterra integral equation epidemiological models [21, 27].

### ACKNOWLEDGMENTS

The authors would like to thank the late Prof. C.I. Christov as well as Prof. M.D. Todorov for the invitation to present this work at the AMiTaNS'12 conference in Varna, Bulgaria. We thank the anonymous referee for comments, which have improved the readability of the paper and the consistency in notation.

The first and third authors were supported by the National Research Foundation of South Africa. The second author acknowledges the support of the TIS Project, funded by the French Ministry of Health and the European Union.
